# Shortening the preparation time of the single prolonged breath-hold for radiotherapy sessions

**DOI:** 10.1259/bjr.20210408

**Published:** 2021-12-21

**Authors:** Michael John Parkes, Stuart Green, Jason Cashmore, Qamar Ghafoor, Thomas Clutton-Brock

**Affiliations:** 1 School of Sport, Exercise & Rehabilitation Sciences, University of Birmingham, Birmingham, United Kingdom; 2 National Institute for Health Research (NIHR)/Wellcome Trust Birmingham Clinical Research Facility, Birmingham, United Kingdom; 3 Hall Edwards Radiotherapy Group, University Hospitals Birmingham NHS Foundation Trust, Birmingham, United Kingdom; 4 Marie Sklodowska-Curie Fellow, Department of Radiation Oncology, University Medical Centre, Amsterdam, Netherlands; 5 Department of Anaesthesia and Intensive Care Medicine, University of Birmingham and University Hospitals Birmingham NHS Foundation Trust, Birmingham, United Kingdom

## Abstract

**Objective:**

Single prolonged breath-holds of >5 min can be obtained in cancer patients. Currently, however, the preparation time in each radiotherapy session is a practical limitation for clinical adoption of this new technique. Here, we show by how much our original preparation time can be shortened without unduly compromising breath-hold duration.

**Methods:**

44 healthy subjects performed single prolonged breath-holds from 60% O_2_ and mechanically induced hypocapnia. We tested the effect on breath-hold duration of shortening preparation time (the durations of acclimatization, hyperventilation and hypocapnia) by changing these durations and or ventilator settings.

**Results:**

Mean original breath-hold duration was 6.5 ± 0.2 (standard error) min. The total original preparation time (from connecting the facemask to the start of the breath-hold) was 26 ± 1 min. After shortening the hypocapnia duration from 16 to 5 min, mean breath-hold duration was still 6.1 ± 0.2 min (*ns vs* the original). After abolishing the acclimatization and shortening the hypocapnia to 1 min (a total preparation time now of 9 ± 1 min), a mean breath-hold duration of >5 min was still possible (now significantly shortened to 5.2 ± 0.6 min, *p* < 0.001). After shorter and more vigorous hyperventilation (lasting 2.7 ± 0.3 min) and shorter hypocapnia (lasting 43 ± 4 s), a mean breath-hold duration of >5 min (5.3 ± 0.2 min, *p* < 0.05) was still possible. Here, the final total preparation time was 3.5 ± 0.3 min.

**Conclusions:**

These improvements may facilitate adoption of the single prolonged breath-hold for a range of thoracic and abdominal radiotherapies especially involving hypofractionation.

**Advances in knowledge:**

Multiple short breath-holds improve radiotherapy for thoracic and abdominal cancers. Further improvement may occur by adopting the single prolonged breath-hold of >5 min. One limitation to clinical adoption is its long preparation time. We show here how to reduce the mean preparation time from 26 to 3.5 min without compromising breath-hold duration

## Introduction

Breath-holding techniques are increasingly used to mitigate respiratory motion during thoracic and abdominal radiotherapy. For instance, during radiotherapy treatment for breast cancer, there are clear benefits of distributing treatment in each treatment fraction over multiple, deep inspiration breath-holds.^1–6^ Strictly, it is better to call these multiple (~10) short (~20 s) breath-holds with air, because we describe below how the same deep inspirations can also be used to achieve prolonged breath-holds of >5 min.^7^

While beneficial, there are a number of reasons why multiple short breath-holds with air are still not ideal. First, they require multiple pauses and resettings between treatment. Secondly, hypofractionation now requires increasing dose delivery and duration of each fraction. Thirdly, treatment is best avoided during the first 15 s of this ~20 s breath-hold,^8,9^ because here there is particularly large settlement movement (up to 1.5 cm) of the diaphragm, pancreas and probably of all internal structures.

Breath-hold duration can, however, be easily prolonged using a combination of preoxygenation and mechanically induced hypocapnia.^9–11^We described how healthy subjects^10–12^ and breast cancer patients^9^ can be trained to deliver such single, or indeed multiple,^13^ prolonged breath-holds safely for >5 min. This is so safe and straightforward that even patients with angina can be trained for such prolonged mechanical hyperventilation.^14^

Using the single prolonged breath-hold for radiotherapy treatment may solve these problems with multiple short breath-holds with air. First, delivering the entire fraction in one breath-hold would eliminate the multiple pauses and resetting. Secondly the 5–10-fold increase in duration facilitates the increased dose delivery of hypofractionation. Thirdly, the increased duration would enable not treating in the first 15 s of the breath-hold, where movement is largest.

But currently, the long preparation time of the prolonged breath-hold is a practical limitation. Here, we describe for trained subjects how to reduce this preparation time. The key preparation components^7,15^ are to

- rest and acclimatize the patient to the ventilator
- increase the oxygen (O_2_) content in the lungs (preoxygenation)
- hyperventilate to lower the partial pressure of carbon dioxide (PCO_2_) to induce a hypocapnia level of 20 mmHg
- maintain this hypocapnia long enough to be effective (to equilibrate PCO_2_ in all extra- and intracellular spaces^16^)

The time taken for preoxygenation cannot be shortened further (and takes only *ca*. 0.5 min) and can be discounted since it is given simultaneously with hyperventilation. We show by how much we can reduce the other components of the preparation time without unduly compromising breath-hold duration.

## Methods

Experiments following the Declaration of Helsinki^17^ were conducted in the NIHR/WTCRF and with approval of the University Hospitals Birmingham R & D team, as described previously.^9–11,13^ We recruited 44 healthy subjects (17 were female) aged 20–25 years old, with no previous experience of breath-holding. Not all performed all experiments because, since we recruited over 7 years, some experiments were complete (needed no more subjects) and technical improvements led to further experiments which were available only to the nine final subjects. Once recruited, no data from any subject were excluded and the exact numbers recruited for each experiment are indicated in the figures.

Subjects listened to music via headphones throughout and could not watch a clock. Subjects lay on a bed, breathing at rest (eupnea) in a supine or semi-recumbent position (depending on their comfort) and were instrumented as described previously to measure systolic blood pressure (sBP), arterial blood oxygen saturation (SpO_2_) and end-expired partial pressure of CO_2_. Subjects were not told what durations they or others achieved until experiments were finished.

In previous work, we recorded a three lead electrocardiogram (ECG).^13^ This is no longer done because hypocapnia has no detectable effect on the ECG, heart rate or heart function even in patients with angina,^14^ nor has breath-holding any such effects in healthy subjects or breast cancer patients.^9–11^

All devices were connected to a programmable CED1401 (Cambridge Electronic Design, Cambridge, England) for data collection and analysis.^9–11,13^ Subjects were instructed to break if breath-holds reached our pre-determined safety limits.^11^

### Training for single prolonged breath-holds

**Figure 1.**
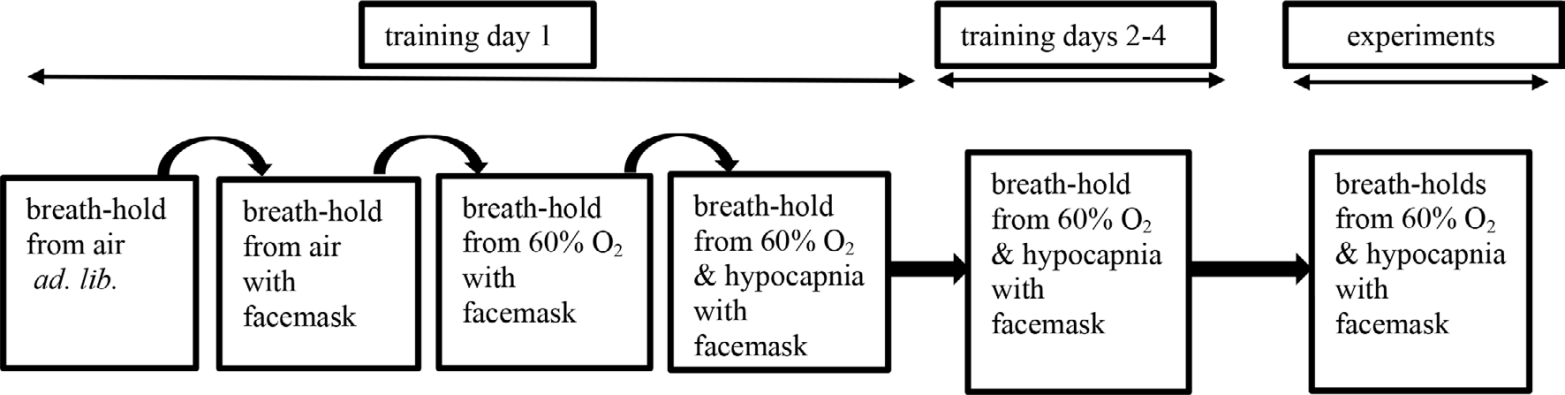
Flow diagram of training and experiments.

All 44 subjects were first trained to deliver the single prolonged breath-hold as follows (see flow diagram in Figure 1).

On day 1, they breath-held from air *ad-lib*., were taught how to breath-hold properly^9–11,13^ and to breathe spontaneously through a facemask connected to the ventilator. (Here, the ventilator mode was “spontaneous”, where subjects still completely controlled their own breathing). They performed a second breath-hold while wearing the facemask (ventilator mode “spontaneous’) while breathing air and a third while breathing 60% O_2_ (again mode “spontaneous).

Subjects were then trained to be mechanically hyperventilated^9–11,13^ using Drager Evita two or Hamilton TI non-invasive mechanical ventilators in “control” mode. Here, the ventilator imposes the hyperventilation pattern and subjects are entirely passive.

They were mechanically hyperventilated with 60% O_2_ (pre-oxygenation) at ~16 breaths.min^−1^. Inflation volume was gradually increased to ~1–2 l (in proportion to body size). This hyperventilation gradually induces hypocapnia, with the induction rate depending on how fast inflation volume is increased. Hypocapnia (a PetCO_2_ level of 20 mmHg) was maintained for 16 min. The ventilator was then switched back to “spontaneous” mode to enable subjects to perform the single prolonged breath-hold. They practiced this single prolonged breath-hold on three subsequent days.

For each subject, we derived mean breath-hold duration from all four practice breath-holds and mean durations of the preparation components from the fourth breath-hold.

### Experiments reducing the preparation time

**Figure 2.**
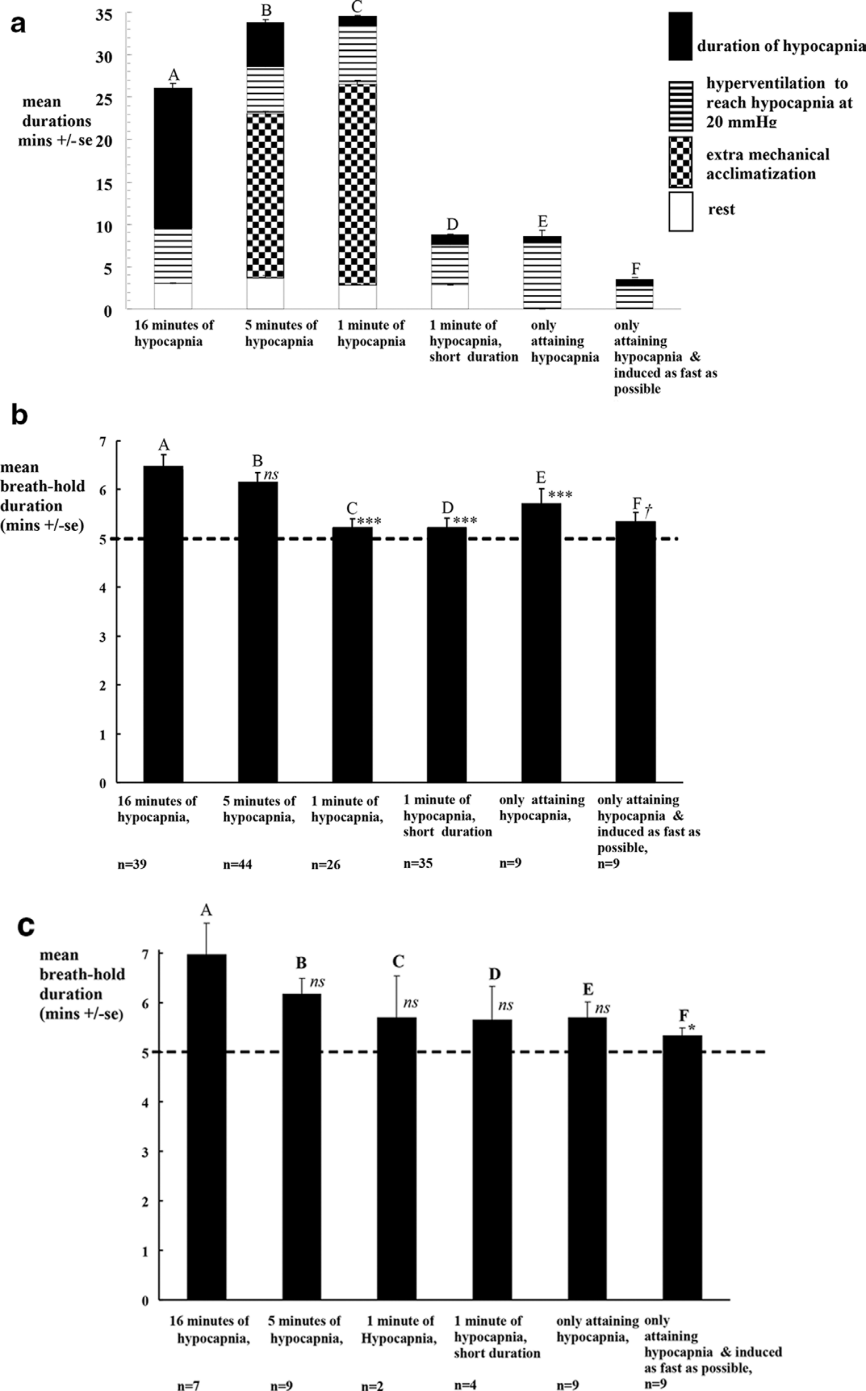
a: Mean + se component durations during mechanical ventilation. Standard errors are indicated and some are too small to be visible. (*N.B.,* Statistical comparisons within figure 2a are inappropriate since durations were deliberately varied). b: Mean + se breath-hold durations in each experimental protocol. The dashed line indicates an arbitrary duration of 5 minutes. n= number of subjects. ns *p*> 0.05 vs. A by paired comparison; †p< 0.05 vs. A by unpaired comparison; *** *p*< 0.001 vs. A by paired comparison. c: Mean + se breath-hold durations in each experimental protocol. The dashed line indicates a duration of 5 min. n= number of the 9 subjects and which experiments they undertook. ns *p*> 0.05 vs. all 39 in A by unpaired comparison. * *p*< 0.05 vs. all 39 in A by unpaired comparison.

Columns A to F in Figure 2a show how our preparation components are derived and how the subsequent five experiments on separate days shortened or removed these components.

Column A shows our original breath-hold methodology,^9–11,13^ where hypocapnia at 20 mmHg was applied for 16 min. The total preparation time was the sum of the durations of rest, mechanical hyperventilation and hypocapnia.

Column B shows reducing the duration of hypocapnia to 5 min. In case shortening the total time spent on the ventilator affected breath-hold duration, we added an extra period, called “mechanical acclimatization”. This was to compensate for the loss of 11 min of hypocapnia in column A. Here, we continued the same mechanical hyperventilation but added CO_2_ to prevent hypocapnia. While its duration had to be at least 11 min, it was arbitrarily increased to 19 min because we anticipated differences between subjects and on different days.

Column C show reducing the duration of hypocapnia to 1 min. The mechanical acclimatization time was proportionately increased by 4 min.

Colum D shows 1 min of hypocapnia with a shorter duration, where the acclimatization period was removed.

Column E shows only attaining hypocapnia, where the rest and 1 min hypocapnia periods were removed. Subjects were mechanically hyperventilated with 60% O_2_ as soon as the facemask was connected and the breath-hold started as soon as a stable level of hypocapnia (20 mmHg) was reached.

Colums F shows only attaining hypocapnia which was induced as fast as possible. We established for each subject the fastest speed with which they were comfortable having inflation volume increased, thus inducing hypocapnia as fast as possible. Again, the breath-hold started as soon as a stable level of hypocapnia (20 mmHg) was reached. Subjects undertook one or two such breath-holds and data is the mean from both.

### Data and statistical analysis

Data were analyzed as described previously.^9–11,13^ Statistical analysis for multiple comparisons was by generalized estimating equations or repeated measures ANOVA with one within subject factor followed by pair-wise contrasts.^9–11,13^ Comparisons were made against mean duration from 60% O_2_ and hypocapnia for all four practice days (figure legends). Significance was taken at *p* < 0.05 (*ns* indicates *p* ≥ 0.05) with two tail tests. Results are expressed as mean ± standard error (se).

## Results

### Statistical analysis

Significant Wald χ_2_ statistics for breath-hold durations were 1548 with 8 degrees of freedom (*p* < 0.001) and for PCO_2_ levels were 400 with 9 degrees of freedom (*p* < 0.001). Significant F value for SpO_2_ was F (2,67)=65 (*p* < 0.001).

### Training sessions for breath-holds with air and with preoxygenation

**Figure 3.**
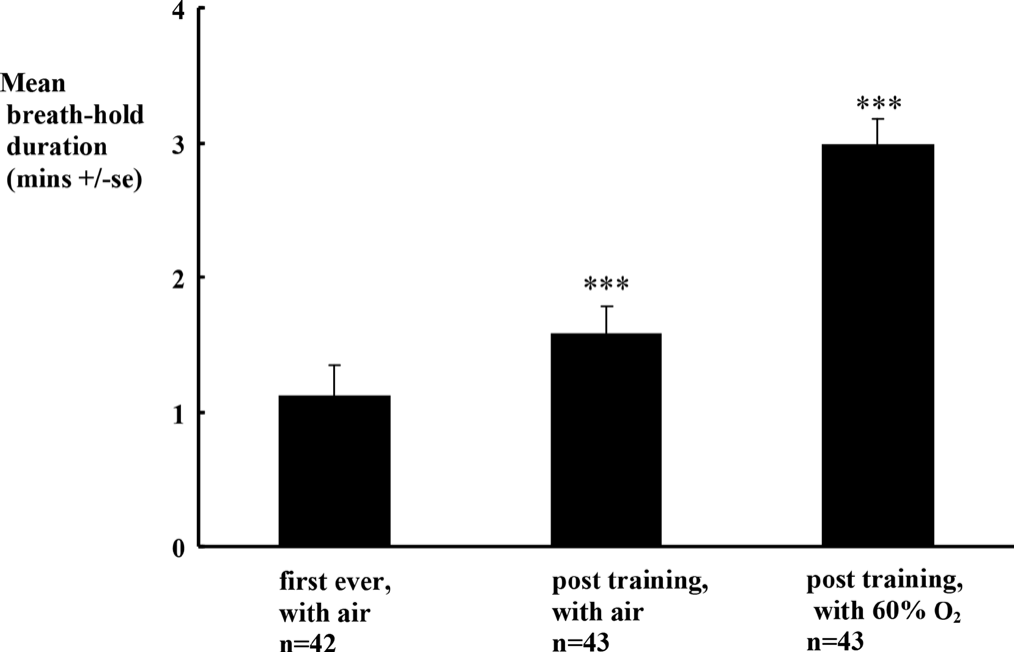
Pre-oxygenation at least doubles breath-hold duration. Mean +se breath-hold durations in each experimental protocol. n= number of subjects. ***p< 0.0001 vs. first ever by paired comparison. e, standard error.

All 44 subjects had normal eupneic heart rate (75 ± 2 b.p.m.), systolic blood pressure (120 ± 3 mmHg), PetCO_2_ (35 ± 1 mmHg) and SpO_2_ levels (98±0 %). Figure 3 shows that on Day 1, their first ever *ad lib.,* mean breath-hold duration with air was 1.1 ± 0.1 min. After training, mean duration with air increased significantly to 1.6 ± 0.1 min (*p* < 0.001), with a mean breakpoint PetCO_2_ level of 44 ± 1 mmHg (*p* < 0.001 *vs*. their mean eupneic PetCO_2_ level) and a mean breakpoint SpO_2_ level of 94±0% (*p* < 0.001 *vs*. their mean eupneic SpO_2_ level).

Spontaneously breathing 60% O_2_ significantly raised their eupneic SpO_2_ to 99±0% (*p* < 0.001). Figure 3 shows that this significantly increased their mean breath-hold duration to 3.0 ± 0.2 min (*p* < 0.001), and hence significantly raised their breakpoint PetCO_2_ level to 50 ± 1 mmHg (*p* < 0.001). Mean SpO_2_ at breakpoint now fell by only a trivial 0.4% (*p* < 0.05* vs*. the eupneic SpO_2_ level with 60% O_2_).

### Shortening the preparation time

Figure 2a shows how preparation was shortened and Figure 2b shows the resulting breath-hold durations.

The total preparation time for our original single prolonged breath-hold (the time from connecting the facemask to both patient and ventilator until the start of the breath-hold) was 26 ± 1 min. Thus, we allow 3 min of rest and it took a mean of 7 ± 1 min for mechanical hyperventilation to lower PetCO_2_ to 20 mmHg (Figure 2a column A). We allowed a 16 min duration of hypocapnia at 20 mmHg (for PCO_2_ equilibration throughout extracellular and intracellular spaces). The mean breath-hold duration achieved was 6.5 ± 0.2 min (Figure 2b column A). Mean PetCO_2_ at breakpoint was 42 ± 1 mmHg.

Shortening only the duration of the hypocapnia to 5 min does not significantly shorten mean breath-hold duration (remaining at 6.1 ± 0.2 min, *ns* see the B columns in Figure 2a & b). Mean total preparation time had risen to 34 ± 1 min because we had deliberately overcompensated for the loss of the 11 min of hypocapnia (by increasing the extra mechanical acclimatization time to 19 min). Our physiological monitoring showed no change in physiological status of the subjects with increased acclimatization. Therefore, neither does increasing the acclimatization time improve breath-hold duration. Neither did this improve CO_2_ equilibration (PetCO_2_ at breakpoint was still 44 ± 1 mmHg, *ns vs* [A]).

Shortening the duration only of hypocapnia to 1 min significantly shortened mean breath-hold duration to 5.2 ± 0.2 min (*p* < 0.001, see the C columns in Figure 2a & b). Mean total preparation time was again 34 ± 1 min. CO_2_ equilibration was impaired as PetCO_2_ at breakpoint was significantly higher *vs* [A], at 47 ± 1 mmHg (*p* < 0.001 and *p* < 0.05* vs*. [B]). Thus, the critical duration of hypocapnia to equilibrate CO_2_ stores is somewhere between 1 and 5 min.

Trained subjects need to spend remarkably little time being mechanically ventilated. Thus, removing the acclimatization period and reducing the mean total preparation time to 9 ± 1 min (3 min of rest, 5 min to lower PetCO_2_ and 1.1 ± 0.1 min. duration of hypocapnia) did not further shorten mean breath-hold duration (still 5.2 ± 0.2 min, see the D columns in Figure 2a & b). Mean PetCO_2_ at breakpoint was 44 ± 3 mmHg (*p* < 0.001 *vs*. [ A]).

Even removing the rest period and maintaining hypocapnia only long enough to convince the operator that it was stable (0.7 ± 0.1 min) is still sufficient for *a* > 5 min breath-hold duration (mean of 5.7 ± 0.3 min)—see the E columns in Figure 2a & b). Yet, CO_2_ equilibration is further impaired (PetCO_2_ at breakpoint rose to 48 ± 3 mmHg (*p* < 0.001)) and total preparation time was 9 ± 1 min.

Finally, a mean breath-hold duration 5.3 ± 0.2 min is still possible just by inducing hypocapnia as fast as possible and breath-holding immediately. Here, the total preparation time was only 3.5 ± 0.3 min (taking only 2.7 ± 0.3 min to lower PetCO_2_). CO_2_ equilibration remained impaired (PetCO_2_ at breakpoint at 50 ± 3 mmHg, *p* < 0.001).

These nine subjects are representative of all subjects, because replotting columns A to D with only these subjects shows the same overall effects (the trends between columns A–D in Figure 2b and c are the same).

Thus, experienced staff can reduce the mean preparation time for our single prolonged breath-hold of >5 min in trained volunteers from 26 to 3.5 min.

## Discussion

The introduction of non-invasive, mechanical ventilation in conscious, unmedicated patients could revolutionize radiotherapy delivery for thoracic and abdominal cancers. This is both by regularizing patient’s breathing pattern for periods of up to 1 h^18–20^ and by enabling them to deliver single,^9^ or multiple^13^ prolonged breath-holds.

Here, we demonstrate how to reduce the preparation time of trained subjects in each radiotherapy session for the single prolonged breath-hold of >5 min from 26 to 3.5 min.

### Training for non-invasive mechanical ventilation for radiotherapy

Non-invasive mechanical ventilation and hypocapnia of conscious, unmedicated patients is safe and inexpensive to apply and simple for therapy radiographers to learn and to deliver. Indeed trained patients may fall into a light sleep during it and have to be roused to breath-hold.

The skill in first training to breath-hold for >5 min is in gradually introducing mechanical ventilation to the patient. If they can breath-hold for >5 min on their first attempt, no further training is necessary. Otherwise, training can be completed outside the radiotherapy clinic in 2–4 sessions over 2–3 days. Patients will then deliver repeated >5 min breath-holds on demand in a radiotherapy setting.^9^

### Effects of shortening the preparation components on breath-hold duration

Our original^9–11,13^ preparation for our >5 min breath-hold takes 26 min (Figure 2a column A), which is impractical in a busy radiotherapy clinic.

We show how the initial rest period (of 3 min) and the time spent being acclimatized (>11 min just being mechanically ventilated) are redundant, (Figure 2a columns B–D). In fact once trained and listening to music, subjects relax remarkably quickly while being mechanically hyperventilated.

Moreover, the 16 min of hypocapnia can be reduced to 0.7 min (Figure 2a column E). This 0.7 min (43 s) represents merely the time it took to convince the ventilator operator that this hypocapnia level was stable and to prepare for the breath-hold. Strictly while still >5 min, this breath-hold duration is significantly shortened because the minimum CO_2_ equilibration time is somewhere between 1 and 5 min (Figure 2b column E). Since 0.7 min is less than the minimum CO_2_ equilibration time of 1–5 min, the resulting PetCO_2_ level at breakpoint is now higher.

Figure 2a & b show too the latitude available if preparation time needs extending (if for instance the patient wanted to stop ventilation briefly to ask a question, or if markers or patient position needed adjustment). Indeed patients can be safely kept hypocapnic for about 1 h.

### Accelerating induction of hypocapnia by fine tuning the ventilator to the patient

Figure 2a & b column F shows how radiographer experience with fine-tuning will accelerate induction of hypocapnia at 20 mmHg as fast, comfortably and safely as possible, to a mean of 3.5 min. The precise settings for mechanical ventilation depend on the patient’s size, resting metabolic rate and comfort. In our subjects, heights ranged between 158 and 190 cm and weights ranged between 51 and 103 kg. Ideally therefore, part of the patient’s initial training period includes the radiographer establishing how fast the optimum ventilation parameters for each patient can be safely applied.

Our ventilator settings (16 breaths.min^−1^ with volumes up to *ca*. 2 l or inflation pressures up to *ca*. 36 cm H_2_O) are greater than the modern lung-protective ventilation strategies recommended for clinical management of acute respiratory distress syndrome.^21^ However, mechanical ventilation over the tens of minutes required for radiotherapy treatment is quite different from the hours or days required for other medical conditions. In fact non-invasive mechanical ventilation of fully conscious patients with intact lungs means that there are negligible risks from the short duration of these settings.

The fastest ever was reached with 2.3 min (still with a breath-hold duration of 5.5 min).

### How representative are these healthy subjects to patients?

We found previously that patients with breast cancer^9^ had single prolonged breath-holds as long as 5.3 ± 0.2 min, and similar physiological responses, to those of equally trained healthy volunteers (5.5 ± 0.5 min^11^). We do not observe further increases in breath-hold duration beyond the initial training period. The healthy volunteers here had the same training regime as we used previously and over the same time period. Their even longer mean breath-hold duration 6.5 ± 0.2 min is not therefore due to their having had more practice. These times will be equally applicable in patients since their handling of CO_2_ is no different.

### Why use a mechanical ventilator to prolong breath-hold duration?

Establishing a preparation time of only 3.5 min begs the question of why use a ventilator? Why not just ask patients to voluntarily hyperventilate with 60% O_2_? Pre-oxygenation does enable breast cancer patients to double mean breath-hold duration, from 42 ± 2 s to 96 ± 0.6 s,^9^ and a similar doubling (to 78 s) was found for patients with lung cancer.^22^ But even when combining pre-oxygenation with voluntarily hyperventilation, it is not possible to achieve breath-hold durations beyond *ca*. 3 min in patients^23,24^ because the physical effort involved also increases CO_2_ production, and hence opposes inducing hypocapnia and shortens breath-hold duration. Furthermore, voluntary hyperventilation is stressful, tiring, requires substantial patient cooperation and the uncontrollable level of hypocapnia achieved can induce paraesthesiae and tetany.^25^

Whereas with mechanical hyperventilation, the radiographer has complete and safe control of patient’s ventilation and PCO_2_ level. Because the patient does nothing, the mean breath-hold duration is much longer (>5 min).

### Advantages of the single prolonged breath-hold for hypofractionation

With hypofractionation, the higher dose per fraction requires more multiple short breath-holds of air per session. We show how the duration of each breath-hold could be prolonged either to 3 min by replacing air with 60% O_2_, or to >5 min with 60% O_2_ and hypocapnia. The chest may naturally deflate too much in 5 min for accurate target irradiation. Yet, two planning CTs during a 5 min breath-hold would indicate how much more time is available to deliver a larger dose in a single breath-hold whilst still optimising target delineation and sparing of organs at risk.

## Conclusions

We show here how the preparation time in each radiotherapy session can be shortened to a mean of 3.5 min and still achieve a mean single prolonged breath-hold duration of 5.3 ± 0.2 min. This improvement may facilitate adoption of this prolonged breath-hold for a range of thoracic and abdominal radiotherapies and for treatments delivered with hypofractionation.
